# Impact of Low-Temperature Storage on the Microstructure, Digestibility, and Absorption Capacity of Cooked Rice

**DOI:** 10.3390/foods11111642

**Published:** 2022-06-02

**Authors:** Hui Li, Bingxiao Liu, Kezia Bess, Zhengxuan Wang, Mingcai Liang, Yan Zhang, Qiong Wu, Lin Yang

**Affiliations:** 1 School of Life Science and Biotechnology, Harbin Institute of Technology, Harbin 150001, China; huili_1024@163.com (H.L.); zhangtyo@hit.edu.cn (Y.Z.); kigo@hit.edu.cn (Q.W.); 2 School of Chemistry and Chemical Engineering, Harbin Institute of Technology, Harbin 150001, China; m18845618140@163.com (B.L.); 18646199358@163.com (Z.W.); hitmcl@163.com (M.L.); 3 Department of Chemistry, Faculty of Natural Sciences, University of Guyana, Turkeyen 999073, Guyana; kezia.bess@uog.edu.gy

**Keywords:** cooked rice, low-temperature storage, microstructure, absorption capacity, digestibility

## Abstract

This study examined the effects of low-temperature storage on the microstructural, absorptive, and digestive properties of cooked rice. Cooked rice was refrigerated and stored at 4 °C for 0.5, 1, 3, 5, and 7 days, as well as frozen and preserved at −20, −40, and −80 °C for 0.5, 1, 3, 5, 7, 14, 21, and 28 days. The results indicated that the stored rice samples generally exhibited a higher absorption capacity for oil, cholesterol, and glucose than the freshly cooked rice. In addition, after storage, the digestibility of the cooked rice declined, namely, the rapidly digestible starch (RDS) content and estimated glycemic index (eGI) decreased, whereas the slowly digestible starch (SDS) and resistant starch (RS) content increased. Moreover, the increment of the storage temperatures or the extension of storage periods led to a lower amylolysis efficiency. Scanning electron microscopy (SEM) analysis indicated that storage temperature and duration could effectively modify the micromorphology of the stored rice samples and their digestion. Moreover, microstructural differences after storage and during simulated intestinal digestion could be correlated to the variations in the absorption capacity and digestibility. The findings from this study will be useful in providing alternative storage procedures to prepare rice products with improved nutritional qualities and functional properties.

## 1. Introduction

As an important cereal grain, rice is a main carbohydrate and energy source for humans worldwide, especially for Asians. There are diverse types of rice available on the market that vary in quality. Rice quality is a comprehensive trait that includes the milling quality (such as roughness ratio, milled rice rate, and head rice rate), the appearance (such as grain size, shape, chalkiness, and transparency), the cooking and eating quality (such as flavor, texture, taste, and gelatinisation temperature), and the nutritional quality (such as micronutrient, starch, protein, and lipid content) [1,2]. Among these, the nutritional quality, which is a typical internal quality indicator, is important for the promotion and maintenance of human health.

As starch is the main constituent of rice, the starch digestion property of cooked rice is an important determinant of its nutritional quality [1]. Normally, rice is consumed after refining and polishing. However, numerous studies have demonstrated that polished rice after cooking induced high postprandial glucose, as it was digested rapidly [3,4,5]. The long-term frequent consumption of rice was always considered to increase the risk of chronic diseases such as hyperglycaemia and diabetes [6,7]. Accordingly, ways to slow the starch digestion rate of cooked rice have attracted intense attention.

In recent years, increasing studies have been conducted to investigate rice starch digestibility [6,8,9,10]. The variations in the grain’s physical properties (gelatinisation, retrogradation, etc.) [11,12] and chemical compositions (amylose, lipid, protein, etc.) [13,14] have been reported to influence rice starch digestibility. Some commonly used processing techniques, such as extrusion cooking [15], thermal processing [16], and post-cooking storage [17], were applied to alter the physical state of rice starch and its digestibility. In particular, the approach of using low-temperature storage after cooking has gained considerable interest, providing an option to modify starch digestion behaviours both in the rate and extent [18]. In addition, with the ever-accelerating pace of life, an increasing number of people prefer pre-cooked rice products, which are stored at cool/freezing temperatures and require only a few minutes to reheat before ingestion [19]. Moreover, attributable to the continuous improvement and application of cryogenic technology and its supporting equipment, low-temperature stored rice products are gradually becoming popular and have promising market potential [20].

Cooked rice is a metastable and non-equilibrium system that undergoes structural alterations during storage [21]. Upon cooling/freezing, the amylose and amylopectin molecules in the gelatinised starch could expel water and realign themselves into more ordered crystalline structures. These changes are mainly related to the retrogradation process, which reduces the susceptibility of starch to digestive enzymes coincident with an increase in the formation of slowly digested starch (SDS) or resistant starch (RS) [22]. As such, low-temperature storage treatments could modify the composition and structural organisation of starchy foods, which might bring about some positive physiological and metabolic effects. Kwaśniewska-Karolak et al. found that the storage of baked goods at low temperatures facilitated starch retrogradation, which ultimately reduced staling-associated reaction rates. Moreover, they also demonstrated that low-temperature storage could extend the period of acceptable quality of baked goods without chemical preservatives [23]. To date, several studies have focused on the physicochemical changes in cooked rice that underwent low-temperature storage [24,25,26]. However, relatively few studies have addressed the starch digestibility and the microstructure (including after storage and throughout the starch digestion process) of low-temperature storage modified cooked rice (especially frozen-stored cooked rice), not to mention the related structure–digestibility interactions.

Besides the occurrence of structural and compositional changes, the processing of low-temperature storage could also influence the functionality of starchy foods. Noteworthily, due to the richness of polysaccharides and dietary fibre in cereal and cereal fractions, an increasing number of studies have focused on investigating their absorption/binding capacity for glucose, oil/fat, cholesterol, and bile acid [27,28,29], in order to explore their potential in vitro hypoglycemic and hypolipidemic effects. In addition, a few studies have also pointed out that the implementation of some process technologies, including extrusion, milling, rolling, shredding, and toasting could help to improve the health potentials of cereals or their fractions [27,30]. In the case of cooked rice grains, although starch polysaccharides are their major components, no study has examined the impact of low-temperature storage conditions on the absorption/binding capacity of such a food matrix.

Increasing demand for convenient foods, coupled with preferences for potential health benefits and the availability of rice in abundance, stimulates the necessity to evaluate the changes that low-temperature storage treatment induces on the absorption capacity and digestibility of cooked rice. In this current study, the starch digestion rates and extents of cooked rice were detected by applying an in vitro-simulated intestinal digestion model. The morphological changes of cooked rice after storage and throughout digestion were determined with scanning electronic microscopy (SEM). The potential capacities of the cooked rice samples to absorb oil, cholesterol, and glucose were investigated. Moreover, the structural features and the absorptive and digestive properties of cooked rice were correlated to elucidate the mechanisms by which the low-temperature storage treatment exerted these effects. The findings of this study will expand the understanding of the microstructural, absorptive, and digestive profiles of low-temperature storage-modified cooked rice and provide valuable insight into the potential applications of low-temperature storage strategies to rice products.

## 2. Materials and Methods

### 2.1. Materials

Milled Japonica rice grains (*Oryza sativa* L. cv. Longjing 26) were kindly provided by the Rice Research Institute of Heilongjiang Academy of Agricultural Sciences (Jiamusi, Heilongjiang, China). The proximate analysis was performed according to AOAC method [31] and indicated that these rice grains contained 11.48% moisture, 77.54% starch (with 18.33% amylose), 7.62% proteins, and 0.91% lipids. The gelatinisation temperature of the rice sample was 69.27 °C. Porcine pancreatic α-amylase (A3176, 10 U/mg) was supplied by Sigma-Aldrich Chemical Co. (St. Louis, MO, USA). Amyloglucosidase from *Aspergillus niger* (A107823, 100,000 U/mL) was obtained from Shanghai Aladdin Biochemical Technology Co., Ltd. (Shanghai, China). d-Glucose Assay Kit (GOPOD format) and Total Cholesterol Assay Kit (COD-PAP format) were purchased from Nanjing Jiancheng Bioengineering Institute (Nanjing, China). All chemical reagents used in this study were of analytical grade.

### 2.2. Rice Cooking

Rice (800 g) was rinsed with water three times, then soaked in 1040 mL distilled water at an ambient temperature for 30 min. The soaked rice was cooked for 30 min, followed by an additional 15 min with heat-preservation mode using an automatic rice cooker (MB-WFS4029, Midea Co., Ltd., Guangzhou, China). The bottom and top layers of cooked rice, as well as the rice adhering to the walls of the cooker, were discarded. The rest was transferred to stainless steel trays for pre-cooling at room temperature, and then the cooked rice samples were packaged separately in polyethylene bags with the same weight and sealed for subsequent low-temperature storage.

### 2.3. Low-Temperature Storage

Packed cooked rice samples were assigned to two storage systems: chilled storage and frozen storage. In the case of chilled storage, the cooked rice samples were stored in a refrigerator at 4 °C for 12 h, 1 d, 3 d, 5 d, and 7 d, whereas for frozen storage, the cooked rice samples were frozen and stored at −20 °C, −40 °C, and −80 °C for 12 h, 1 d, 3 d, 5 d, 7 d, 14 d, 21 d, and 28 d. After storing for the different periods, the sealed bags of cooked rice were removed and immediately freeze-dried using a freeze dryer (SCIENTZ-10N/A, Ningbo Scientz Biotechnology Co. Ltd., Ningbo, China) at 0.1 mbar, −55 °C for 24 h. Then, the resultant freeze-dried samples were ground and passed through a 500 μm sieve cassette (Pulverisette19, Fritsch, Munich, Germany) for later investigation. Freshly cooked rice was used as control.

### 2.4. Determination of Absorption Capacity

#### 2.4.1. Oil Absorption Capacity

Oil absorption capacity (OAC) was determined according to Lin et al. [32], with some modifications. Cooked rice samples (1 g, dry basis) were dispersed in 10 mL soybean oil. Then, the mixture was mixed thoroughly and placed in a water bath at 37 °C for 1 h, stirring for 30 s every 10 min. After centrifugation at 1600× *g* for 15 min, the supernatant fluid was discarded, and the precipitate was weighed. The OAC was calculated as follows:

Oil absorption capacity (g/g) = (W_2_ − W_1_)/W_1_, (1)

where W_1_ and W_2_ referred to the weight of cooked rice samples before and after oil absorption, respectively (g).

#### 2.4.2. Cholesterol Absorption Capacity

For the determination of cholesterol absorption capacity (CAC), 0.5 g cooked rice samples on dry weight basis were combined with 25 mL of 60% ethanol (containing cholesterol of 100 μg/mL). The solution was mixed well and incubated (37 °C, 6 h) with continuous oscillation. The mixture was centrifuged at 3000× *g* for 15 min and the cholesterol concentration in the supernatant was determined using the Total Cholesterol Assay Kit. The cholesterol absorption capacity was measured as follows:

Cholesterol absorption capacity (%) = (G_0_ − G)/G_0_, (2)

where G_0_ was the initial cholesterol concentration (100 μg/mL = 0.259 mmol/L) and G was the cholesterol concentration in the supernatant after 6 h (mmol/L).

#### 2.4.3. Glucose Absorption Capacity

Glucose adsorption capacity (GAC) of cooked rice samples was determined according to Ou et al. [33], with some modifications. Cooked rice samples (0.5 g, dry basis) were added to 25 mL of glucose solution (10 mmol/L). The slurry was mixed thoroughly and then incubated in a shaking water bath at 37 °C for 6 h. After centrifugation at 3000× *g* for 15 min, the glucose content in the supernatant was determined using the d-Glucose Assay Kit. The amount of glucose absorbed was calculated using the following formula:

Glucose absorbed (μmol/g) = [(10 − G) × V]/W,(3)

where 10 was the initial glucose content (mmol/L), G was the glucose concentration in the supernatant after 6 h (mmol/L), V was the volume of solution (25 mL), and W was the weight of rice samples (0.5 g).

### 2.5. In Vitro Starch Digestibility

A small intestinal-simulated in vitro digestion model was applied according to previous work with some modifications [9]. Briefly, cooked rice samples and white bread (reference) containing 100 mg of total starch (TS), which was measured according to the procedure of Goñi et al. [34], were dispersed in 20 mL of deionised water and then incubated in a water bath at 37 °C for 15 min. After equilibration, six glass balls (6 mm diameter) and simulated intestinal fluid (10 mL, containing 8228 U α-amylase and 204 U amyloglucosidase), were added, followed by incubation in a shaking water bath at 37 °C and 200 strokes/min for 3 h. Aliquots (1 mL) were withdrawn throughout the digestion process at 10, 20, 30, 45, 60, 90, 120, and 180 min and heated immediately in a boiling water bath for 10 min to inactivate enzymes. Subsequently, the reaction systems were centrifuged at 3000× *g* for 10 min and the glucose contents of the supernatants were determined using the d-Glucose Assay Kit.

The starch hydrolysis rate was converted from the amount of glucose dissolving out of the hydrolysis. Starch digestibility curves were then plotted as hydrolysis rate versus reaction time. The contents of different starch fractions of rapidly digested starch (RDS), SDS, and RS were determined based on the glucose concentrations at different hydrolysate times [35].

### 2.6. Estimation of Glycemic Index (eGI)

The starch digestibility curves were fitted to a first-order rate equation [*C* = *C*_∞_ (1 − exp(–*k*t))] [34], wherein *C*, *C*_∞_, and *k* were the hydrolysis percentage, the equilibrium hydrolysis percentage, and the kinetic constants, respectively. Among these, *C*_∞_ and *k* were estimated by using Origin Pro software version 2019. The hydrolysis index (HI) was obtained by dividing the area under the hydrolysis curve (AUC) of each rice sample by that of white bread. The estimated glycemic index (eGI) was calculated using the formula eGI = 39.71 + 0.549 HI [34].

### 2.7. Microstructural Analysis

A scanning electron microscope (SEM) was utilised to visualise the morphological changes of cooked rice after storage and during the in vitro digestion process. The freshly cooked and stored rice samples were collected and pretreated as described in Section 2.3. The digested rice samples were collected from the digestion reactors at 20, 60, 120, and 180 min, immediately soaked in liquid nitrogen, and then freeze-dried. Subsequently, the freeze-dried samples (freshly cooked, stored, and digested) were spread evenly onto the surface of aluminium plates and coated vertically with a thin film of gold-palladium to make them electrically conductive in a sputter coater (JFC-1100, Joel Ltd., Tokyo, Japan). The obtained specimens were then scanned and imaged with an SEM analyser (TM-1000, HITACHI, Tokyo, Japan) operating at an accelerating voltage of 20 kV and magnifications of 5000× (for freshly cooked and stored rice samples) and 2400× (for digested rice samples).

### 2.8. Statistical Analysis

All assays were implemented in triplicate and resulting data were expressed as mean values ± standard error. Two-way analysis of variance (ANOVA) followed by Tukey’s HSD test were conducted on means comparison and *p* < 0.05 was considered to indicate a significant difference. The SPSS 26.0 software (Chicago, IL, USA), Origin Pro 2019 (Northampton, MA, USA), and R software (R version 3.6.3) were used for statistical computations, mapping, and correlation analysis.

## 3. Results and Discussion

### 3.1. Microstructure Changes after Storage

**Figure 1:**
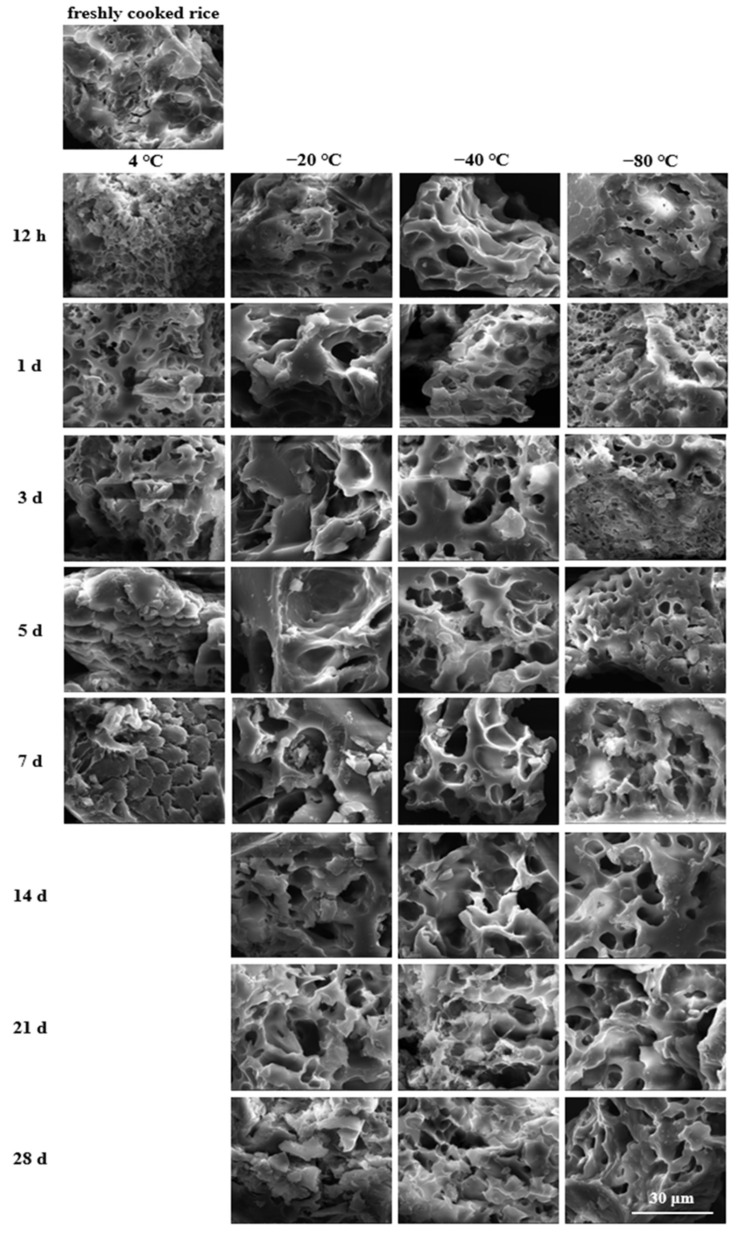
Scanning electron microscopic images of cooked rice samples at different storage temperatures and periods (5000×, bar = 30 μm).

The cooked rice matrices revealed varying microstructures corresponding to the storage conditions (Figure 1). The fresh-cooked rice (control) showed indentations and slight cracks on the surface, whereas the refrigerated and frozen rice samples treated with different storage temperatures and periods exhibited more micropores.

In the case of cold storage, the rice samples displayed a coarse and discontinuous honeycomb-like structure during the first 3 days, and the pores became larger and more irregular with the extension of the storage time. This phenomenon indicated that the cooked rice had a greater disconnection of hydrogen bonds between the water and starch molecules due to the retrogradation process during cooling storage [36]. As a result, the interactions between the starch molecules increased, and the water gradually expelled from the gelatinised starch granules leading to the formation of a porous structure after freeze-drying [36,37,38]. As the storage period increased to 5 and 7 days, the appearance of the cooked rice became more continuous and compact with less porosity, which signified the extensive aggregation of starch molecules through hydrogen bonds. This aggregation, which might be a typical characterisation of retrogradation progression, occurred during refrigerated storage because the amylose and amylopectin chains in the gelatinised rice grains could spontaneously reassociate into more ordered structures [39].

Differing from the refrigerated cooked rice, most frozen cooked rice samples developed porous structures due to the formation of ice crystals during storage and exhibited a marked dependence on the storage temperature: the higher the temperature, the larger the pore size. Likewise, Song et al. revealed that the pore sizes of freeze-dried cooked rice stored at −70 °C were smaller than those of the samples stored at −20 °C and −10 °C [40]. The difference in the porous structures may depend on the size distribution of ice crystals, and the latter is also closely related to the freezing rate [41,42]. Typically, a lower freezing temperature is associated with faster freezing and consequently takes starchy foods through the zone of maximum ice crystal formation quicker and creates a higher nucleation rate [43,44,45]. Accordingly, at the initial and middle storage times, the rice stored at −80 °C displayed a dense structure with finer porosity, whereas at −20 and −40 °C the rice samples exhibited relatively unconsolidated structures with larger cavities due to a narrow thermal gradient.

In addition, for the rice samples stored at −20 °C after 14 d or at −40 °C after 21 d, the intensity of the agglomeration and weak porous architecture of the granules were apparent as the storage time increased, probably as a result of the progression of starch retrogradation. This recrystallisation process led to the gradual disruption of the starch granules into small crystallising particles. Consequently, distinctive phase segregation into starch-rich and starch-deficient regions was created due to the lower water diffusion and more flexible starch component mobility [21,45]. Furthermore, as can be seen, a few irregularly shaped pores were embedded in the starch-rich phase matrix. Such phenomenon could not be clearly observed in the −80 °C stored rice samples, but samples stored for 28 d presented a relatively loose appearance with less porosity. This suggests that a lower storage temperature could be more effective in inhibiting phase separation behaviour during starch retrogradation.

### 3.2. In Vitro Absorption Capacity

#### 3.2.1. Oil Adsorption Capacity (OAC)

**Figure 2:**
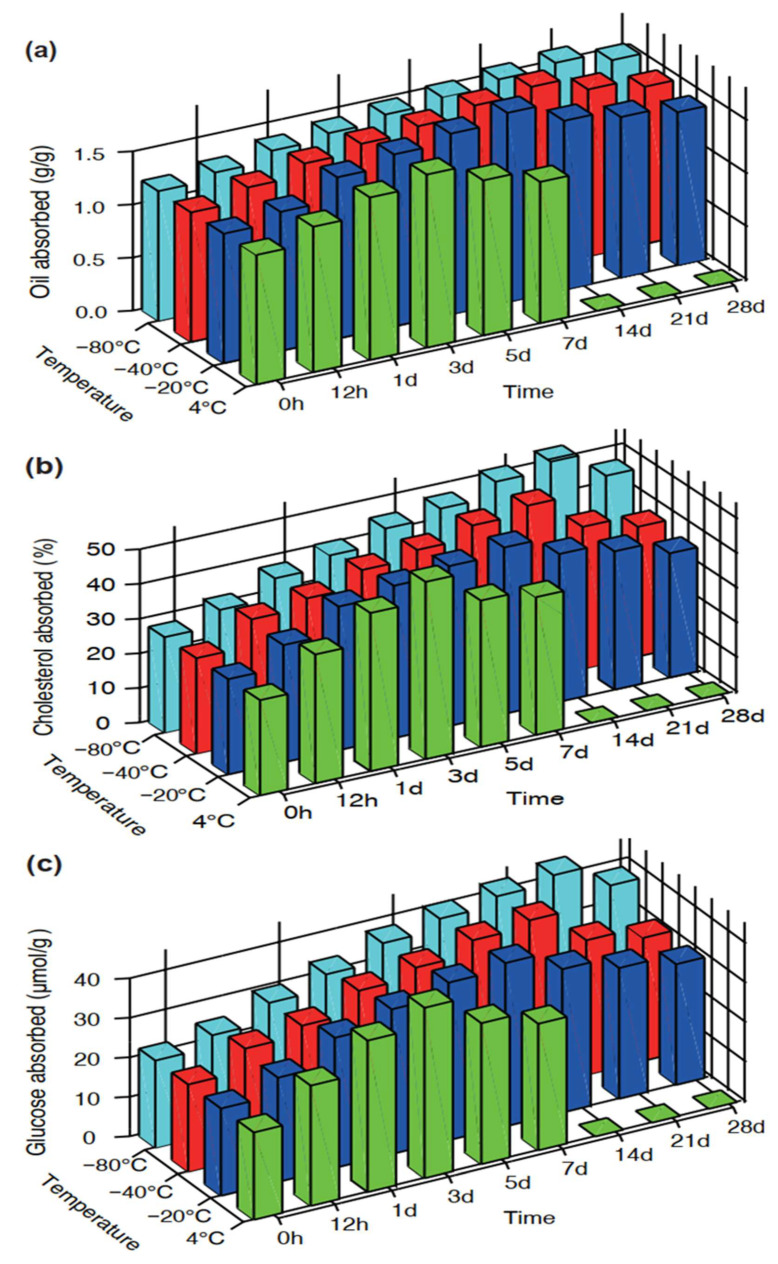
In vitro absorption capacities of various cooked rice samples for oil (**a**), cholesterol (**b**), and glucose (**c**). Values are the means ± SEM (*n* = 3).

According to Figure 2a, most of the stored rice samples showed significantly (*p* < 0.05) higher OAC values than their fresh counterpart, except for several frozen rice samples that exhibited slight increments after storing for 12 h. In addition, the oil absorption capacities of the 4, −20, −40, and −80 °C stored rice samples were in the range of 1.33~1.63, 1.31~1.78, 1.34~1.71, and 1.28~1.62 g/g, respectively, suggesting a greater oil absorption potential for cooked rice samples stored at −20 and −40 °C with respect to those stored at 4 and −80 °C. From the results, the stronger OAC may be due to the porous granule structure and the increased lipophilic tendency after low-temperature storage treatment. According to Figure 1, −20, and −40 °C stored rice samples had many large and deep pores, which could increase the total pore volume and the contact area with oil, thereby contributing to more exposure of the oleophilic groups and the enhancement in the entrapment ability of oil drops [46,47].

#### 3.2.2. Cholesterol Absorption Capacity (CAC)

As observed in Figure 2b, subjecting cooked rice to low-temperature storage modified its ability to quench cholesterol to a higher extent. Specifically, all the studied stored rice samples were characterised by higher CAC values in comparison with the fresh sample, and the increment rates were 35.21~85.70%, 23.01~74.30%, 28.06~83.48%, and 15.90~94.85% (*p* < 0.05) for the rice samples stored at 4, −20, −40, and −80 °C, respectively. Herein lies the vital influence of storage temperature since higher absorbing performances are shown at 4 and −80 °C, which may be attributed to their greater pore density and aperture ratio. That is, according to the micromorphology described in Figure 1, a comparatively smaller size structure with finer porosity was formed on the surfaces of the 4 and −80 °C stored rice samples, which might contribute to an increased specific surface area, thus providing more binding sites for cooked rice samples to bind to cholesterol molecules [48]. Contrarily, at storage temperatures of −20 and −40 °C, the relatively larger and deeper pore structures presented on the granule surface might interfere with the bonding strength to cholesterol, thus decreasing affinity and even leading to the desorption effect at the final centrifugation process [29].

#### 3.2.3. Glucose Adsorption Capacity (GAC)

The GAC responses of the cooked rice samples are shown in Figure 2c. The results showed that the GAC values increased noticeably with significant differences (*p* < 0.05) among the stored rice samples when compared with the freshly cooked rice. That is, the low-temperature storage treatment seemed to help to enhance the glucose absorption capacity of cooked rice. Moreover, in agreement with the tendency recorded in the cholesterol absorption, the rice samples stored at 4 and −80 °C were found to have a relatively greater potential for absorbing glucose. This may be attributed to the higher pore density and aperture ratio that appeared on the surfaces of the 4 and −80 °C stored rice samples, which may promote the exposure of more functional groups inside the cooked rice matrix, thus binding and entrapping more free glucose and reducing glucose concentration in the media [49].

### 3.3. Relationship between Absorption Capacity, Microstructural Characteristics, and Low-Temperature Storage Conditions

In this present study, for different cooked rice matrixes, their absorption capacity mainly relied on their surface structural characteristics, as well as the nature of the absorbed substances [50]. The greater oil-binding potential of the −20 and −40 °C stored rice samples may be attributed to their relatively larger-sized pores, as well as the liquid lipid property of soy oil. That is, soy oil drops could penetrate more easily into larger pores [51]. For this current study, however, in the case of the cholesterol and glucose absorption capacities, the cholesterol and glucose existed as solute molecules. In this context, cooked rice samples with comparatively smaller porous structures could become more accessible for glucose or cholesterol to react [52]. These would be the main reasons for the higher binding potentials of the 4 and −80 °C stored rice samples, which even presented honeycomb-like structures during the initial storage period.

Importantly, from Figure 2, an interesting trend was noticed where the oil, cholesterol, and glucose absorption capacities first rose and then fell as the storage duration extended. Such trend variations could be largely attributed to the microstructural changes in the stored rice samples. According to Figure 1, after cooking, low-temperature storing, and freeze-drying, the rice samples’ surfaces became porous and rough and showed dimensional network structures during the initial storage period, which would provide channels in which oil, cholesterol, or glucose could get entangled. With the increase in the storage time, the loosened structures with larger and deeper pores could leave more open space for entrapping or binding the oil, cholesterol, or glucose, thus generating a higher absorption efficiency. However, with a further extension of the storage time, the previous porous structures were gradually damaged due to the progression of starch retrogradation, which would be responsible for the decreasing absorption efficiency during the end of the storage period.

To date, the link between the absorption capacity and microstructural characteristics of cooked rice is rarely described in the literature and no studies have focused on the specific role of low-temperature storage conditions in modulating the absorption capacity. The results from our study demonstrate the significant role of low-temperature storage conditions in affecting the oil, cholesterol, and glucose absorption capacities by regulating the microstructural properties of the cooked rice matrix. Improvements in living standards have been accompanied by an increased intake of fat, cholesterol, and glucose. As such, ways to reduce the excess lipids and glucose in the body have received increasing attention. In this current study, flours from low-temperature stored cooked rice have been confirmed to have potential in their in vitro hypolipidemic and hypoglycaemic effects. This signifies a promising application for these stored rice samples as a functional food to reduce the amount of glucose and lipids available in the intestinal lumen.

### 3.4. In Vitro Starch Digestibility

#### 3.4.1. In Vitro Starch Digestion Profile and Kinetics

**Figure 3:**
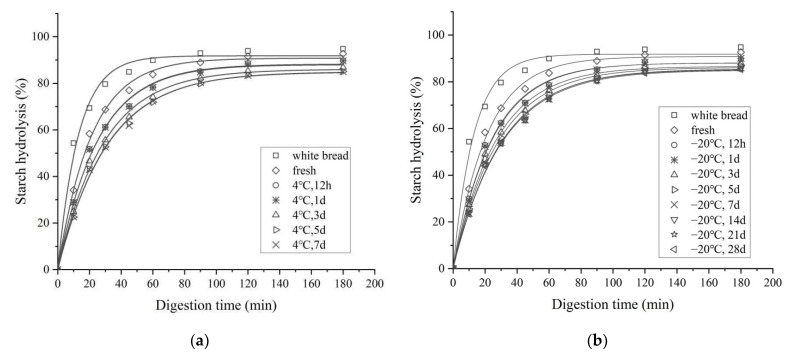

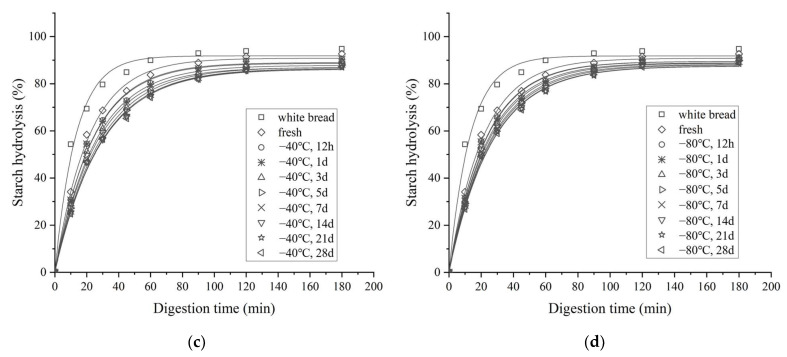
Starch hydrolysis profiles of cooked rice under different storage temperatures and periods. (**a**) 4 °C; (**b**) −20 °C; (**c**) −40 °C; (**d**) −80 °C.

The hydrolysis time curves obtained for white bread and all cooked rice samples are plotted in Figure 3. The digestograms of all the cooked rice samples showed similar digestibility patterns. Specifically, the digestibility rates increased rapidly during the early stages of the 0~60 min period but slowed down thereafter and reached a plateau after about 120 min during the simulated small intestinal digestion process. Compared with the white bread and freshly cooked rice, the stored rice samples decreased the starch hydrolysis degree as early as in the initial digestion phase and subsequently maintained this tendency. Similar results were also reported by Rosin et al. [53] and Sáyago-Ayerdi et al. [54], who studied different food products stored at low temperatures. Accordingly, a negative influence of low-temperature storage on digestion ease was concluded.

As shown in Table 1, the high correlation coefficients (*R*^2^ ≥ 0.995) indicated that the starch digestion of all the rice samples followed first-order behaviour. The values of *C*_∞_ and *k* provided a good fit for the experimental data. In this context, the stored rice samples displayed evidently lower *C*_∞_ and *k* values compared to their fresh counterpart (*p* < 0.05), which was consistent with the above results of the hydrolysis profiles. Additionally, the *C*_∞_ and *k* values were found to be negatively related to the storage temperature and duration, and higher storage temperatures/longer storage periods elicited decreased *C*_∞_ and *k* values. In this study, the reduced *k* values under refrigerated/frozen storage might be mainly attributed to starch retrogradation and impaired digestibility by amylase, which depended strongly on the storage temperature. That is, a lower temperature induces faster retrogradation because nucleation and crystal growth are favoured [43].

#### 3.4.2. Starch Fractions

**Figure 4:**
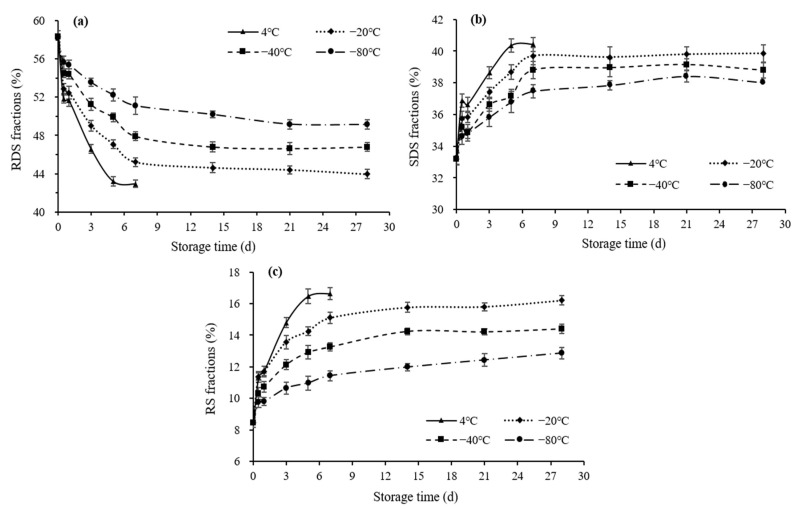
Starch fractions of cooked rice under different storage temperatures and periods. (**a**) RDS; (**b**) SDS; (**c**) RS. RDS, rapidly digested starch; SDS, slowly digested starch; RS, resistant starch. Error bars show the standard error of the mean (*n* = 3).

As shown in Figure 4, the increase in the SDS and RS contents due to a reduction in the RDS content was observed in all the stored rice samples in comparison with the fresh sample, which agreed with the tendency recorded for the hydrolysis rates. Meanwhile, more pronounced changes in starch fractions were observed as the storage temperature increased. Moreover, in terms of the overall trend in the variations in the starch fractions, their contents changed rapidly in the first 12 h and then decreased/increased continually with gradual slower rates over the storage time. In this instance, it may be inferred that in vitro starch digestibility was affected more easily in the first 12 h than in the other storage periods. Interestingly, a similar observation had been reported by Ren et al., who studied the starch digestion properties of frozen foxtail millet products and found that the first day of frozen storage had a stronger influence on starch digestibility than the remaining days [18].

On storage, such changes in starch fractions could imply retrogradation occurrence and microstructural alterations, which could affect the susceptibility to enzymatic hydrolysis. Starch retrogradation during storage results in the formation of a perfect crystalline structure that increases the RS content or an imperfect crystalline structure that increases the SDS content [55]. Based on the data obtained in this study, although a reduction in RDS subjected to retrogradation caused a simultaneous increase in RS and SDS, the RS content exhibited a higher increment extent than the SDS content. Thus, with low- temperature storage, the retrogradation process tended to form a perfect crystalline structure and an increased RS content in cooked rice. Similar results were also mentioned by Rosin et al., who observed a significant increase in the RS amount of cooked whole or polished rice under −20 °C for 30 days [53]. In addition, Ayimbila et al. reported an increase in the RS content when cooked jasmine rice was stored at 4 °C and −20 °C for 3 days [55]. Furthermore, given that starch retrogradation is manifested by fast amylose recrystallisation and slow amylopectin recrystallisation [18], it is, therefore, reasonable to infer that the amylose retrogradation was mainly responsible for the relatively rapid change in the contents of the starch fractions after 12 h of storage. The amylopectin rearrangement occurred at the expense of a fall in the RDS content, which implied a slow process of change in the internal structure of the cooked rice and that could be the primary reason for the relatively slow modification of starch during further storage.

### 3.5. Estimated Glycemic Index

As seen in Table 2, the HI and eGI for white bread (as reference) and freshly cooked rice (as control) were 100 and 93.57, and 94.61 and 91.08, respectively. By comparison, the HI and eGI of all the stored rice samples reduced significantly (*p* < 0.05), varying between 81.82~91.31 and 84.63~89.84, respectively. Notably, the results also confirmed that a higher storage temperature/longer storage period had a more prominent effect on the eGI of cooked rice, which coincided with the digestion characteristics indicated above.

In food processing, most starch-based products are usually subjected to heating and cooking. Therefore, it is more practical to investigate the GI values of starchy foods after cooking [56]. Overall, the results of this study showed that for all the rice samples, the eGI values were more than 84, indicating that they belonged to the high GI food category. However, the eGI values of the stored rice samples were obviously lower than that of the fresh sample, which might be attributed to their decreased starch digestibility and increased RS content. As such, a low-temperature storage treatment was proven to have the potential to relieve the rapid rise of the postprandial glycemic index, especially with the refrigeration treatment.

### 3.6. Pearson Correlation Analysis

The Pearson correlation coefficients for the relationship among the starch digestibility parameters of the stored cooked rice samples are presented in Table 3.

In our case, cooling and freezing treatments were found to have relatively subtle impact on starch digestibility. As can be seen, the eGI, *C*_∞_, and *k* values of cooked rice showed a positive correlation with the RDS content, but a negative correlation with the RS and SDS contents. The results obtained, therefore, allow the inference that the starch fractions of cooked rice were susceptible to storage conditions and might play a role in influencing the glycaemic response and starch susceptibility to amylolysis. Although the findings of this current study are in agreement with several studies in the literature [53,54,56], the specific mechanistic basis for this phenomenon has still not been studied in detail. Nonetheless, understanding the dynamics of water and ice and the starch retrogradation behaviour of stored cooked rice might provide a noteworthy insight. That is, as the storage time increased, water migration and evaporation occurred upon cooling and ice crystal growth and aggregation occurred upon freezing, producing gradual changes in the internal structure of the cooked rice. The interactions of the amylose-amylose, amylopectin-amylopectin, and amylose-amylopectin chains could be facilitated within the damaged structure, leading to greater starch recrystallisation and more RS and SDS formation [57], resulting in further limitations in enzyme accessibility and a reduction in eGI values. Therefore, this current study might provide valuable insights for researchers for designing rice products with lower digestibility, thus gaining nutritional improvements and health benefits.

### 3.7. Microstructural Changes during In Vitro Digestion

**Figure 5:**
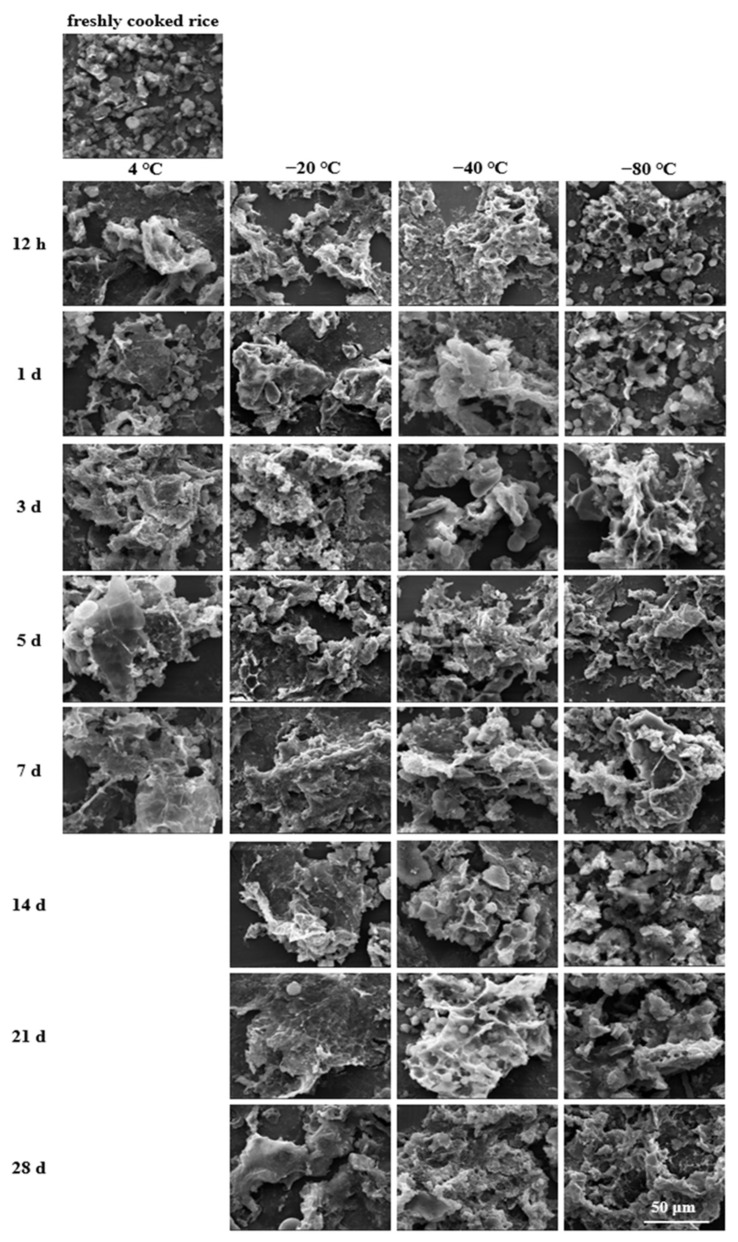
Microstructural changes of fresh and stored rice samples digested in simulated intestinal digestion for 20 min (2400×, bar = 50 μm).

**Figure 6:**
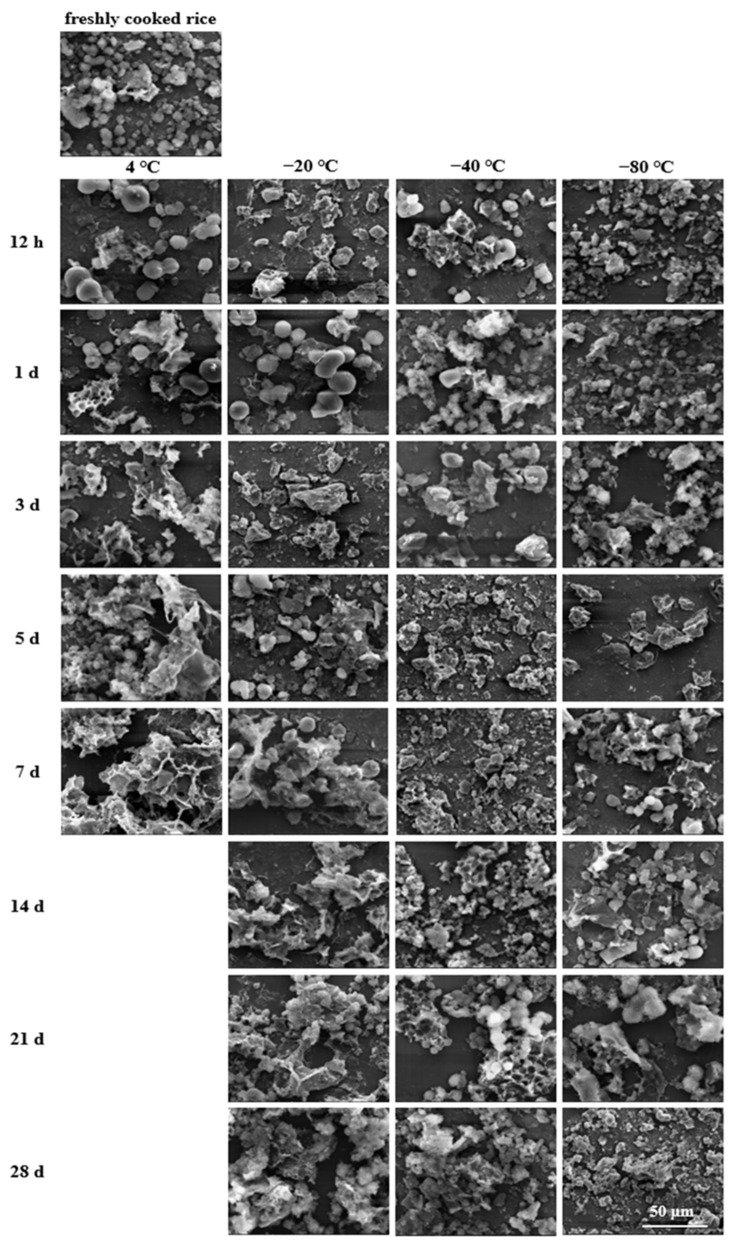
Microstructural changes of fresh and stored rice samples digested in simulated intestinal digestion for 60 min (2400×, bar = 50 μm).

**Figure 7:**
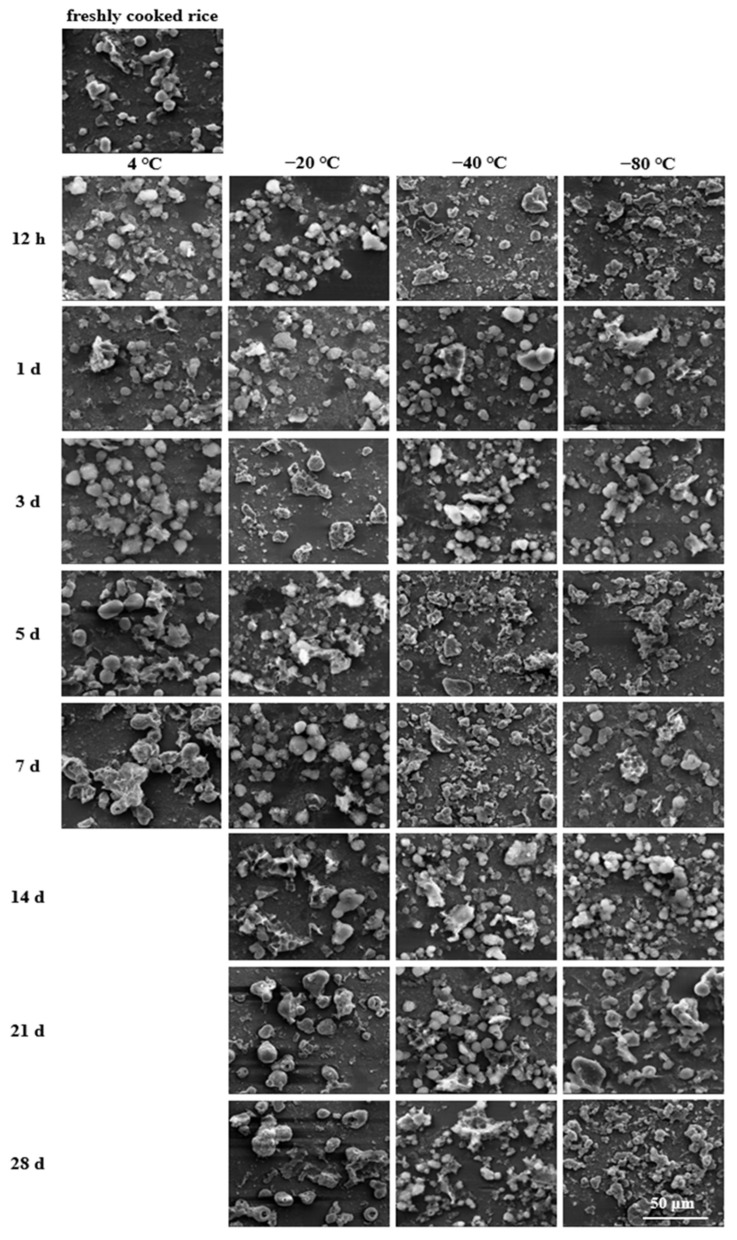
Microstructural changes of fresh and stored rice samples digested in simulated intestinal digestion for 120 min (2400×, bar = 50 μm).

**Figure 8:**
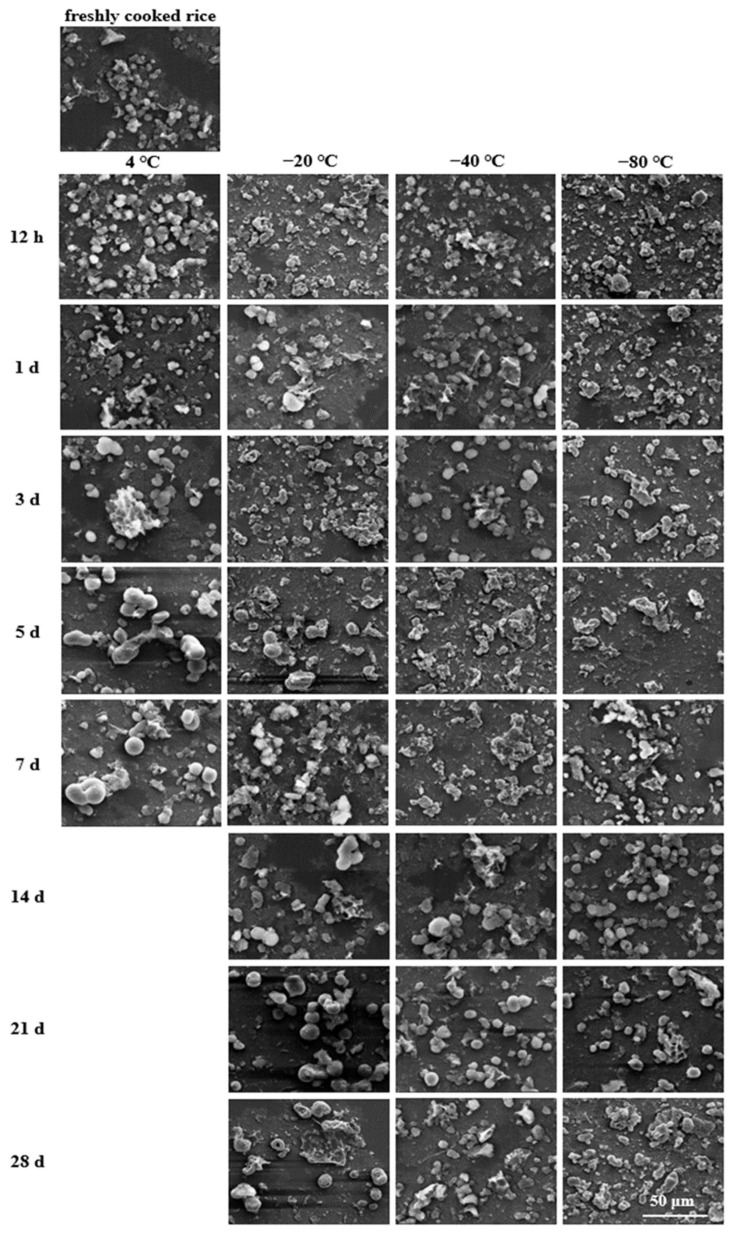
Microstructural changes of fresh and stored rice samples digested in simulated intestinal digestion for 180 min (2400×, bar = 50 μm).

To further analyse the impact of low-temperature storage, the microstructural changes of the stored rice samples during simulated in vitro digestion as well as the counterpart fresh sample were examined using SEM (Figure 5, Figure 6, Figure 7 and Figure 8). As digestion proceeded, the external surface microstructures of all the cooked rice samples were gradually destructed and broken up into smaller forms due to the actions of both chemical and physical forces. Thus, the residue size was gradually reduced implying the progressive digestion of starch from the periphery to the particle core.

After simulated intestinal digestion for 20 min, as shown in Figure 5, it appeared that the digesta surface was coarser and fluffier than that of the undigested cooked rice, which was attributed to shaking and enzymatic hydrolysis. Noticeably, some enzyme-etching plaques, depressions, and fallen fragments occurred on the sample surface, consistent with the features of partial starch digestion. For the freshly cooked rice, the residue structure had many nubs and fragments suggesting better accessibility for enzyme digestion. By comparison, the residue of the refrigerated and frozen cooked rice mainly showed dimensional lamella structures, which were the remnants of starch that were not easily broken down by enzymes. In addition, concerning the effects of low- temperature storage conditions, it was confirmed that the digesta presented larger lamellar residue with a more compact periphery as the storage time extended. The micrographs also revealed more tiny holes or pores and wrinkles on the residual surfaces as the storage temperature decreased.

After additional digestion of 60 min, hydrolysis occurred and the morphology of the digested rice changed significantly. As seen in Figure 6, most rice samples were broken up into smaller forms with chunk-like and irregular-shaped structures. In the fresh sample, the SEM displayed the appearance of granular and fragmented residues. However, with the extension of the storage periods, it was seen that the residues were gradually dominated by larger pieces, and some agglomerated into starch masses with a more compact periphery. In addition, the number of small pieces seemed to be higher in the rice samples which were stored at a lower temperature, indicating better diffusion of digestive juices in these samples, which made them more susceptible to further hydrolysis.

As the digestion progressed further, it was found that the residue of the cooked rice samples consisted mainly of much smaller pieces after digestion for 120 min (Figure 7), signifying extensive hydrolysis of some remaining digestible starch due to the amylase enzymatic action. Thus, the size of the residues became smaller, promoting higher digestion rates and extents. However, such enzymatic action seemed to be weakened during the digestion period of 120~180 min, since the residual rice samples exhibited slight decreases in size and distribution density at the end of the digestion time of 180 min (Figure 8). These microstructural features correlated well with the insignificant changes in the starch digestion rates between 120 min and 180 min, indicating the nearly maximum hydrolysis of amylase on the starch in the cooked rice after 120 min of digestion.

### 3.8. Relationship among Low-Temperature Storage Condition, Microstructure and Digestibility

Generally, the storage temperature and period, as two important quality-determining factors, are believed to be involved in modulating the microstructural and digestive properties of starchy foods. However, the specific role of low-temperature storage conditions in regulating these properties of cooked rice, and the relationships or interactions between the micromorphology of stored rice samples and their digesta and the in vitro starch digestion rate and extent have rarely been studied so far. Thus, a systematic study of the microstructural characteristics and starch digestibility of cooked rice warrants special concern.

In this current study, for all the cooked rice samples, their microstructural properties after storage and during digestion seemed to be closely related to the degree of starch digestion. On the one hand, according to the SEM morphologies before digestion (Figure 1), more amorphous voids and some honeycomb-like structures developed on the stored rice samples during the initial and middle storage periods. The starch granules’ surfaces could be totally hydrolysed, whereas further contact between digestive enzymes and the inner starch would be impeded by the porous structure, especially by the honeycomb structure, hence causing a hindrance to enzymatic hydrolysis [58,59]. Moreover, the visualised rearrangement of starch molecules occurring at the end of the storage period might possibly lead to a significant decrease in starch hydrolysis. This is because such morphological structures indicated stronger starch retrogradation degrees and could act as barriers against enzymes involved in starch hydrolysis. Therefore, the lower amylolysis rate and extent of stored rice samples with respect to their fresh counterpart were in consonance with those micromorphology changes.

On the other hand, allied to the enzymatic action and mechanical shaking, the microstructural properties of the digested rice were altered (Figure 5, Figure 6, Figure 7 and Figure 8). When the storage temperature was lower, the digesta structure seemed to be damaged to a higher degree. Alternatively, the digesta morphology tended to display a more connected structure when the storage period was longer. Finally, after simulated small intestinal digestion, relatively bigger pieces remained as the storage temperature increased or the time extended. These observations concurred with the lower *k* and eGI values and the higher SDS and RS contents of rice samples that underwent a higher storage temperature or a longer storage period. Collectively, the microstructural features after storage and during the simulated intestinal digestion could explain why the cooked rice samples stored at a higher temperature or for a longer period showed lower digestion rates, as well as equilibrium starch hydrolysis. The findings of this study revealed that low-temperature storage conditions played a significant role in regulating the morphologies of the stored rice samples and their digesta, which could in turn affect the in vitro digestion rate.

## 4. Conclusions

This study elucidated how low-temperature storage processing influenced the absorption capacity and digestion characteristics of cooked rice samples by inspecting the microstructural changes after storage and throughout the starch digestion process. Through the usage of in vitro models, the results demonstrated that refrigerated and frozen storage temperatures and periods collaboratively modulated the absorption and digestion behaviours of cooked rice samples. Compared to freshly cooked rice, the stored rice samples exhibited lower RDS contents and eGI values, higher SDS and RS contents, as well as stronger absorption capacities for oil, cholesterol, and glucose, which positioned them as noteworthy vehicles for improving human health. Furthermore, the structure–property relationships indicated the significant role of the structural basis in controlling low-temperature storage-induced modifications in the absorption capacities and digestibility of the cooked rice samples. Nevertheless, this is a preliminary study which provides fundamental data for the rational use of low-temperature storage methods to develop rice products with an associated absorption capacity and digestibility. It is desirable that further investigations be conducted to reveal the structure–property relationships in greater detail and explore the potential application of low-temperature storage processing. Firstly, only SEM was used in this current study. More advanced techniques such as confocal laser scanning microscope, micro-CT, etc., should be combined to improve the understanding of how the microstructure of the refrigerated/frozen cooked rice modulates its absorption and digestion performances. Secondly, only the absorption and digestion properties were investigated in the present research. More physicochemical properties can be studied to comprehensively understand the influence of low-temperature storage treatment on cooked rice. Thirdly, since only in vitro models were applied in this paper, in vivo studies are needed to confirm the health benefits and physiological effects of refrigerated/frozen cooked rice. Fourthly, only one rice genotype was evaluated in this current study. More rice varieties could be employed to investigate their responses to low-temperature storage treatment and provide more evidence on the potential application of low-temperature storage processing.

## Figures and Tables

**Table 1:** Effect of storage temperatures and periods on the hydrolysis kinetics of cooked rice samples.

Parameters	Storage Time (Days)	Storage Temperature			
		4 °C	−20 °C	−40 °C	−80 °C
***R*** **^2^**	0	0.9968	0.9968	0.9968	0.9968
	0.5	0.9959	0.9958	0.9962	0.9963
	1	0.9960	0.9957	0.9956	0.9960
	3	0.9970	0.9965	0.9962	0.9963
	5	0.9979	0.9971	0.9967	0.9972
	7	0.9975	0.9966	0.9969	0.9967
	14		0.9976	0.9971	0.9971
	21		0.9973	0.9975	0.9972
	28		0.9980	0.9964	0.9974
***C*** **_∞_ (%)**	0	92.69 ± 0.43 ^A,c^	92.69 ± 0.43 ^A,d^	92.69 ± 0.43 ^A,c^	92.69 ± 0.43 ^A,c^
	0.5	89.67 ± 0.72 ^A,b^	89.68 ± 0.45 ^A,c^	90.49 ± 0.51 ^A,b^	91.17 ± 0.31 ^A,b^
	1	89.60 ± 0.67 ^A,b^	90.06 ± 0.56 ^A,c^	90.35 ± 0.46 ^A,b^	91.13 ± 0.33 ^A,b^
	3	86.82 ± 0.69 ^A,a^	88.03 ± 0.32 ^A,b^	89.53 ± 0.34 ^B,b^	90.67 ± 0.28 ^C,b^
	5	85.10 ± 1.06 ^A,a^	86.99 ± 0.69 ^AB,ab^	87.93 ± 0.31 ^B,a^	89.92 ± 0.53 ^C,ab^
	7	85.04 ± 0.91 ^A,a^	86.03 ± 0.58 ^A,a^	88.27 ± 0.28 ^B,a^	89.47 ± 0.30 ^C,a^
	14		85.80 ± 0.72 ^A,a^	87.76 ± 0.41 ^B,a^	89.24 ± 0.42 ^C,a^
	21		85.47 ± 0.85 ^A,a^	86.92 ± 0.52 ^A,a^	88.99 ± 0.37 ^B,a^
	28		85.40 ± 0.78 ^A,a^	87.15 ± 0.39 ^A,a^	88.70 ± 0.55 ^B,a^
***k* × ** **10** **^−^** **^2^** ** (min** **^−^** **^1^** **)**	0	4.72 ± 0.08 ^A,c^	4.72 ± 0.08 ^A,d^	4.72 ± 0.08 ^A,d^	4.72 ± 0.08 ^A,d^
	0.5	3.94 ± 0.12 ^A,b^	4.12 ± 0.07 ^AB,c^	4.28 ± 0.06 ^B,c^	4.40 ± 0.07 ^B,c^
	1	3.95 ± 0.07 ^A,b^	4.05 ± 0.04 ^A,c^	4.26 ± 0.08 ^B,c^	4.35 ± 0.05 ^B,c^
	3	3.47 ± 0.08 ^A,a^	3.75 ± 0.06 ^B,b^	3.94 ± 0.07 ^B,b^	4.15 ± 0.04 ^C,b^
	5	3.22 ± 0.06 ^A,a^	3.54 ± 0.05 ^B,ab^	3.85 ± 0.05 ^C,b^	4.04 ± 0.04 ^D,b^
	7	3.20 ± 0.11 ^A,a^	3.33 ± 0.09 ^A,a^	3.62 ± 0.06 ^B,a^	3.91 ± 0..02 ^C,a^
	14		3.35 ± 0.03 ^A,a^	3.50 ± 0.08 ^A,a^	3.84 ± 0.06 ^B,a^
	21		3.31 ± 0.08 ^A,a^	3.54 ± 0.05 ^B,a^	3.76 ± 0.05 ^C,a^
	28		3.32 ± 0.04 ^A,a^	3.49 ± 0.09 ^AB,a^	3.74 ± 0.09 ^B,a^

Means ± SEM (*n* = 3). Values followed by different capital letters in the same row denote significant differences (*p* < 0.05). Values followed by different lower-case letters in the same column denote significant differences (*p* < 0.05). *R*^2^, correlation coefficient; *C*_∞_, equilibrium hydrolysis percentage; *k*, kinetic constant.

**Table 2:** Effect of storage temperatures and periods on the estimated glycemic index of cooked rice samples.

Parameters	Storage Time (Days)	Storage Temperature			
		4 °C	−20 °C	−40 °C	−80 °C
**HI**	0	93.57 ± 0.21 ^A,d^	93.57 ± 0.21 ^A,d^	93.57 ± 0.21 ^A,e^	93.57 ± 0.21 ^A,d^
	0.5	88.54 ± 0.17 ^A,c^	88.95 ± 0.28 ^A,c^	90.41 ± 0.19 ^B,d^	91.31 ± 0.19 ^C,c^
	1	88.30 ± 0.22 ^A,c^	88.71 ± 0.14 ^A,c^	90.04 ± 0.23 ^B,d^	91.14 ± 0.16 ^C,c^
	3	84.29 ± 0.19 ^A,b^	86.09 ± 0.44 ^B,b^	88.02 ± 0.16 ^C,c^	89.91 ± 0.40 ^D,b^
	5	81.83 ± 0.31 ^A,a^	84.71 ± 0.33 ^B,b^	86.76 ± 0.28 ^C,b^	89.30 ± 0.25 ^D,b^
	7	81.82 ± 0.26 ^A,a^	83.10 ± 0.25 ^B,a^	85.89 ± 0.19 ^C,ab^	88.38 ± 0.39 ^D,ab^
	14		82.93 ± 0.18 ^A,a^	84.90 ± 0.35 ^B,a^	87.78 ± 0.24 ^C,a^
	21		82.51 ± 0.22 ^A,a^	84.79 ± 0.40 ^B,a^	87.06 ± 0.31 ^C,a^
	28		82.47 ± 0.15 ^A,a^	84.50 ± 0.51 ^B,a^	86.84 ± 0.44 ^C,a^
**eGI**	0	91.08 ± 0.37 ^A,d^	91.08 ± 0.37 ^A,d^	91.08 ± 0.37 ^A,d^	91.08 ± 0.37 ^A,c^
	0.5	88.32 ± 0.28 ^A,c^	88.54 ± 0.42 ^AB,c^	89.35 ± 0.22 ^B,c^	89.84 ± 0.18 ^B,b^
	1	88.19 ± 0.23 ^A,c^	88.41 ± 0.38 ^AB,c^	89.14 ± 0.12 ^B,c^	89.75 ± 0.36 ^B,b^
	3	85.99 ± 0.17 ^A,b^	86.97 ± 0.33 ^B,b^	88.03 ± 0.36 ^C,b^	89.07 ± 0.24 ^D,b^
	5	84.64 ± 0.42 ^A,a^	86.21 ± 0.25 ^B,b^	87.34 ± 0.29 ^C,b^	88.74 ± 0.39 ^D,ab^
	7	84.63 ± 0.35 ^A,a^	85.33 ± 0.18 ^A,a^	86.87 ± 0.34 ^B,ab^	88.23 ± 0.15 ^C,a^
	14		85.24 ± 0.15 ^A,a^	86.32 ± 0.19 ^B,a^	87.90 ± 0.32 ^C,a^
	21		85.01 ± 0.21 ^A,a^	86.26 ± 0.28 ^B,a^	87.51 ± 0.24 ^C,a^
	28		84.98 ± 0.26 ^A,a^	86.10 ± 0.32 ^B,a^	87.38 ± 0.33 ^C,a^

Means ± SEM (*n* = 3). Values followed by different capital letters in the same row denote significant differences (*p* < 0.05). Values followed by different lower-case letters in the same column denote significant differences (*p* < 0.05). HI, hydrolysis index; eGI, estimated glycemic index.

**Table 3:** Pearson correlations among the starch digestibility parameters for stored cooked rice.

Parameters	*k*	*C*_∞_	HI	eGI	RDS	SDS
*C*_∞_	0.9745					
HI	0.9919	0.9927				
eGI	0.9919	0.9927	1.0000			
RDS	0.9964	0.9841	0.9957	0.9957		
SDS	–0.9880	–0.9491	–0.9686	–0.9686	–0.9858	
RS	–0.9809	–0.9919	–0.9965	–0.9965	–0.9894	0.9510

## Data Availability

Data is contained within the article.
